# MicroRNA-34b/c suppresses uveal melanoma cell proliferation and migration through multiple targets

**Published:** 2012-03-01

**Authors:** Feng Dong, Dinghua Lou

**Affiliations:** The First Affiliated Hospital of College of Medicine, Zhejiang University, Hangzhou, China

## Abstract

**Purpose:**

MicroRNAs (miRNAs) are endogenously expressed, small noncoding RNAs that inhibit gene expression by binding to target mRNAs. Recent studies have revealed that miRNAs function as tumor suppressors or oncogenes. In the present study, we investigated the role of miRNA-34b/c in uveal melanoma.

**Methods:**

Real-time reverse transcriptase polymerase chain reaction (RT-PCR) was performed to detect the expression level of miR-34b/c in uveal melanoma cells and primary samples. Subsequently, uveal melanoma cell proliferation was examined by the MTS (3-[4,5-dimethylthiazol-2-yl]-5-[3-carboxymethoxyphenyl]-2-[4-sulfophenyl] -2H-tetrazolium, inner salt) assay, clone formation assay, and flow cytometry. Cell apoptosis was measured by caspase3/7 assay. Cell migration was evaluated by transwell migration assay. The target of miR-34b/c was predicted by bioinformatics and validated by luciferase assay. In addition, the effect of miR-34b/c on c-Met, cell cycle-related proteins, v-akt murine thymoma viral oncogene homolog (Akt) and extracellular signal-regulated kinase (ERK) pathway was determined by western blotting.

**Results:**

miR-34b/c expression, which was dramatically decreased in uveal melanoma cells and clinical samples, can be upregulated by doxorubicin and epigenetic drugs. The transfection of miR-34b/c into uveal melanoma cells leads to a significant reduction in cell growth and migration. miR-34b/c caused cell cycle G_1_ arrest rather than the induction of apoptosis. Met proto-oncogene (*c-Met*) was identified as a target of miR-34b/c in uveal melanoma cells. Furthermore, miR-34b/c was confirmed to downregulate the expression of c-Met, p-Akt, and cell cycle–related proteins by western blotting.

**Conclusions:**

Our results demonstrate that both miR-34b and miR-34c act as tumor suppressors in uveal melanoma cell proliferation and migration through the downregulation of multiple targets.

## Introduction

MicroRNAs (miRNAs) are endogenously expressed, non-protein-coding RNA molecules with a length of 18–26 nucleotides [1]. These small RNAs directly bind the specific complementary sequences at the target mRNA (mRNA) 3′ untranslated regions (3′ UTRs), negatively regulating the translation and stability of target mRNAs. Lin-4 was the first identified miRNA, and is crucial in regulating the development of *Caenorhabditis elegans* [2]. After this discovery, increasing evidence has indicated that miRNAs are important regulators in diverse processes such as cell proliferation, differentiation, development, and tumorigenesis [3-5]. Aberrant expression of miRNAs can lead to oncogenesis with the enhancement of cell proliferation and metastatic potential [6,7]. Some miRNAs are found to act as oncogenes or tumor suppressor genes. For example, previous studies have revealed that let-7 functions as a tumor suppressor in lung cancer and cutaneous melanoma. The decrease of let-7 in lung carcinoma is linked to upregulation of the *Ras* oncogene [8]. In another report, miR-1 and miR-206 were found to inhibit rhabdomyosarcoma cell proliferation and migration by targeting met proto-oncogene (*c-Met*) [9], which is an important oncogene in many types of human cancers, including lung cancer, hepatocellular carcinoma, and rhabdomyosarcoma. Recently, the miR-34 family was found to be involved in the *p53* tumor suppressor gene effector network, as direct targets of p53 [10-18]. These studies imply that miRNAs play key roles in tumorigenesis through the regulation of cell proliferation, apoptosis, and migration.

In cases of uveal melanoma, which is the most common primary intraocular malignancy in adults, there is no effective treatment for patients with metastasis [19]. Thus, further investigation of uveal melanoma would be helpful to provide novel approaches for clinical therapy. Recent research has linked miRNAs to the development of uveal melanoma. For example, miR-34a and miR-137 have been demonstrated to be involved in the tumorigenesis of uveal melanoma [20,21]. Until now, however, the role of miRNAs in uveal melanoma remains largely unknown.

In the present study, we aimed to investigate the role of miR-34b/c in uveal melanoma. First, we demonstrate that miR-34b/c was downregulated in uveal melanoma cells and clinical samples. The expression of miR-34b/c was upregulated by doxorubicin (DOX) and epigenetic drugs. Furthermore, the introduction of miR-34b/c into tumor cells led to the inhibition of growth through cell cycle G_1_ arrest, instead of the induction of apoptosis. In addition, miR-34b/c inhibited cell migration. We also provide evidence that *c-Met* was a target of miR-34b/c, and miR-34b/c decreased endogenous c-Met, phosphorylated v-akt murine thymoma viral oncogene homolog (p-Akt), cyclin-dependent kinase (CDK) 4, and CDK6 protein levels in uveal melanoma cells. In summary, our study demonstrates that miR-34b/c can function as a tumor suppressor in uveal melanoma cell proliferation and migration.

## Methods

### Cell culture and clinical samples

The human uveal melanoma cell line SP6.5 was isolated from Caucasian patients with primary choroidal melanoma and grown in Dulbecco modified Eagle’s media (DMEM; Invitrogen, Carlsbad, CA) supplemented with 10% fetal bovine serum (FBS; Hyclone, Logan, UT) and incubated at 37 °C in a humidified incubator containing 5% CO_2_, as described [22]. The human primary melanocytes were isolated and cultured as previously described [23]. Briefly, the uveal stromal tissues were isolated from donor eye under the dissecting microscope. The uveal melanocytes were isolated from the uveal stromal segments by trypsin and collagenase disaggregation. Then the primary isolated melanocytes were grown in F12 medium (Invitrogen) supplemented with 10% FBS and incubated at 37 °C in a humidified incubator containing 5% CO_2_.Five human uveal melanoma specimens were obtained from patients treated at the First Affiliated Hospital, Zhejiang University (Hangzhou, China), with documented informed consent in each case. All studies and procedures involving human tissue were performed in compliance with the Helsinki Declaration, and approved by the Zhejiang University Ethics Committee.

### Cell proliferation assay

SP6.5 cells were plated at 3×10^3^ cells per well in 96-well plates (Costar, High Wycombe, UK). For each well, 50 nM of miR-34b/c mimic molecule (Ambion, Austin, TX) or a negative control (Ambion) was transfected into cells using Lipofectamine 2000 (Invitrogen). After 24 h culture, cell proliferation was measured using the CellTiter 96 Aqueous MTS (3-[4,5-dimethylthiazol-2-yl]-5-[3-carboxymethoxyphenyl]-2-[4-sulfophenyl]-2H-tetrazolium, inner salt) assay (Promega, Madison, WI) according to the manufacturer’s instructions. Briefly, the CellTiter 96 AQueous One Solution Reagent was added to each well and incubated at 37 °C for 3 h. Cell proliferation was determined by measuring the absorbance at 490 nm using a microtiter plate reader (Molecular Devices, Sunnyvale, CA).

### Flow cytometry analysis

SP6.5 cells were transfected with 50 nM of miR-34b/c mimic or a negative control. After 48 h, the cells were collected, washed with PBS, and stained with propidium iodide. The stained cells (1×10^5^) were then analyzed for DNA content with a flow cytometer (FACScaliber; Becton-Dickinson, Franklin Lakes, NJ).

### Caspase activity assay

Apoptosis in SP6.5 cells was assessed using the Caspase-Glo 3/7 Assay kit (Promega) according to the manufacturer’s instructions. SP6.5 cells were seeded in 96-well plates and transfected with 50 nM of miR-34b/c mimic or negative control. Cells were then treated with or without doxorubicin (1 μg/ml) for 48 h. The cells were lysed and incubated with the caspase substrate for 2 h, followed by reading by a microtiter plate reader (Molecular Devices, Sunnyvale, CA).

### Transwell migration assays

SP6.5 cells were grown to ~70% confluence and transfected with 50 nM miR-34b/c mimic or a negative control. After 24 h, the cells were harvested by trypsinization and washed once with PBS (Invitrogen). To detect cell migration, 8 mm pore size culture inserts (Transwell; Costar, High Wycombe, UK) were placed into the wells of 24-well culture plates, separating the upper and lower chambers. In the lower chamber, 400 μl of DMEM containing hepatocyte growth factor (HGF; 20 ng/ml; R&D Systems, Minneapolis, MN) was added. Then, 1×10^5^ cells were added to the upper chamber. After 24 h of incubation at 37 °C with 5% CO_2_, the number of cells that had migrated through the pores was quantified by counting five independent visual fields under the microscope (Zeiss, Oberkochen, Germany) using a 20× objective. Cells were stained with hematoxylin and eosin for observation.

### miR-34b/c target prediction

TargetScan was conducted for miR-34b/c target prediction. TargetScan predicts miRNA targets by searching for the presence of conserved 8-mer and 7-mer sites that match the seed region of each miRNA [24]. The possible potential target mRNAs, as well as the potential binding sites of miR-34b/c, were predicted by the TargetScan program.

### Luciferase reporter assays

The Luciferase reporter vectors were constructed as previously reported [20]. Briefly, Human c-Met 3’UTR was amplified by PCR, using human genomic DNA as template. The PCR product was cloned into pMIR-REPORT vector (Ambion).  The recombinant plasmid was named pLuc-MET 3’UTR. The pLuc-MET 3’UTR Mutant plasmid was generated by changing the seed region sequence from CACUGCC to GUGACGG, using QuickchangeXL Mutagenesis Kit (Stratagene, LaJolla, CA).SP6.5 cells were cotransfected with 0.4 μg of firefly luciferase reporter vector and 0.02 μg of the control vector, pRL-SV40 (Promega), using Lipofectamine 2000 (Invitrogen) in 24-well plates (Costar). Each transfection was performed in four wells. For each well, 50 nM of miR-34b, miR-34c mimic, or a negative control was cotransfected with the reporter constructs. Luciferase assays were performed 24 h after transfection using the Dual Luciferase Reporter Assay System (Promega). The *Renilla* luciferase activity was used to normalize the firefly luciferase activity.

### Western blot analysis

SP6.5 cells (1×10^5^) were seeded and grown in DMEM with 10% FBS in 60 mm dishes for 24 h. After transfection, the cells were washed with PBS and subjected to lysis in a lysis buffer (50 mM/l Tris-Cl, 1 mM/l EDTA, 20 g/l sodium dodecyl sulfate, 5 mM/l dithiothreitol, 10 mM/l phenylmethylsulfonyl fluoride). Protein lysates (50 μg each) were separated by 10% sodium dodecyl sulfate –PAGE, then electrotransferred to nitrocellulose membranes. The membranes were blocked in the buffer containing 5% nonfat milk in PBS with 0.05% Tween-20 for 2 h, and incubated overnight with primary antibody at 4 °C. The membranes were washed twice with PBS containing 0.05% Tween-20, then incubated with peroxidase-conjugated secondary antibodies and developed with an electrogenerated chemiluminescence detection kit (Pierce, Rockford, IL). Glyceraldehyde-3-phosphate dehydrogenase (GAPDH) was used as a loading control. All the antibodies, including c-Met, total extracellular signal-regulated kinase 1/2 (ERK1/2), phosphorylated ERK1/2, total Akt, phosphorylated Akt, cyclin-dependent kinase 4 (CDK4), CDK6, and phosphorylated retinoblastoma 1 (Rb) were from Cell Signaling Technology (Beverly, MA).

### Statistical analysis

All data are shown as the mean±standard error of the mean. Differences between groups transfected with miR-34b/c and a negative control were analyzed using the Student *t* test. Statistical significance was accepted at p<0.05.

## Results

### MicroRNA-34b/c expression was downregulated in uveal melanoma cells and specimens

**Figure 1:**
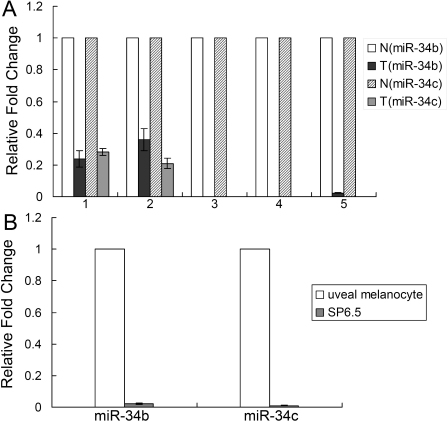
MicroRNA-34b/c expression was downregulated in human uveal melanoma cell line and clinical samples. **A**: Real time reverse transcriptase (RT)-PCR analysis was performed to detect the expression of miR-34b/c in clinical samples. The value for miR-34b/c in normal uveal tissue was set at 1, and the relative amounts of miR-34b/c in tumor tissues were shown as fold induction. Both miR-34b and miR-34c were dramatically decreased in five specimens as compared with normal tissues. N: normal uveal tissue; T: tumor tissue. **B**: The expression of miR-34b/c was measured by real time RT–PCR in uveal melanoma cell line SP6.5, as well as the primary uveal melanocytes. miR-34b/c was expressed in uveal melanocytes but dramatically decreased in uveal melanoma cells. U6 snRNA was used as an internal control.

To investigate whether miR-34b/c was involved in the tumorigenesis of uveal melanoma, we first examined miR-34b/c expression levels in primary samples. Real-time reverse transcriptase (RT)- polymerase chain reaction (PCR) analysis was performed to detect miR-34b/c expression in five specimens of uveal melanoma. The adjacent uveal tissue was used as normal control in each case. As a result, miR-34b/c expression was dramatically decreased in specimens 1, 2, and 5, and undetectable in specimens 3 and 4, in contrast to normal tissues (Figure 1A). We also compared the expression of miR-34b/c in uveal melanoma cell line SP6.5 and primary uveal melanocytes by real-time RT–PCR. Consistent with the results from primary samples, miR-34b/c was expressed in uveal melanocytes, but was dramatically decreased in the SP6.5 cell line (Figure 1B). These results indicate that miR-34b/c expression is frequently downregulated in human uveal melanoma.

### MicroRNA-34b/c expression was upregulated in uveal melanoma cells treated with DOX or epigenetic drugs

**Figure 2:**
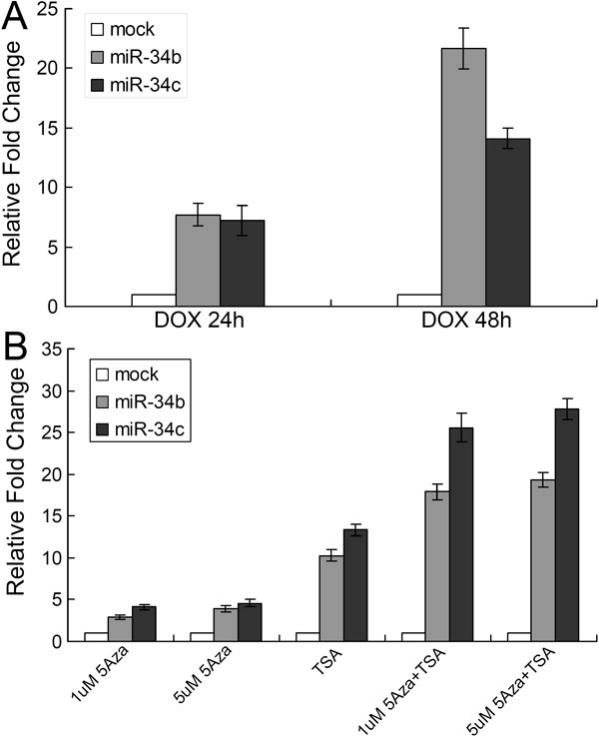
MicroRNA-34b/c was upregulated by doxorubicin and epigenetic drugs. **A**: Real time reverse transcriptase (RT)-PCR analysis was performed to detect the expression of miR-34b/c in uveal melanoma cell line SP6.5, after treatment with DOX for 24 h and 48 h. **B**: SP6.5 cells were treated with 5-aza-dC at 1 μM or 5 μM alone, TSA (100 ng/ml) alone, or combinations of both. miR-34b/c expression level was determined by Real-time RT–PCR. The value for miR-34b/c in SP6.5 cells without any treatment was set at 1, and the relative amounts of miR-34b/c in cells treated with drugs were shown as fold induction. U6 snRNA was used as an internal control.

Treatment of tumor cells with DOX (doxorubicin) or inhibitors of DNA methyltransferases and/or histone deacetylase suppresses cell growth by activating multiple tumor suppressor genes [25-27]. We detected the expression levels of miR-34b/c in SP6.5 cells treated with either DOX, 5-aza-dC (a DNA hypomethylating agent), and/or TSA (a histone deacetylase inhibitor). Expression of miR-34b/c increased after treatment with DOX at 24 h, with maximal induction at 48 h (Figure 2A). Similarly, miR-34b/c was also upregulated by 5-aza-dC or TSA (Figure 2B). Furthermore, the effect of drug combination seems to be additive on miR-34b/c expression (Figure 2B). Overall, these results demonstrated that the expression of miR-34b/c in uveal melanoma cells can be affected by DOX and epigenetic drugs.

### MicroRNA-34b/c inhibited uveal melanoma cell proliferation and induced G_1_ cell cycle arrest

**Figure 3:**
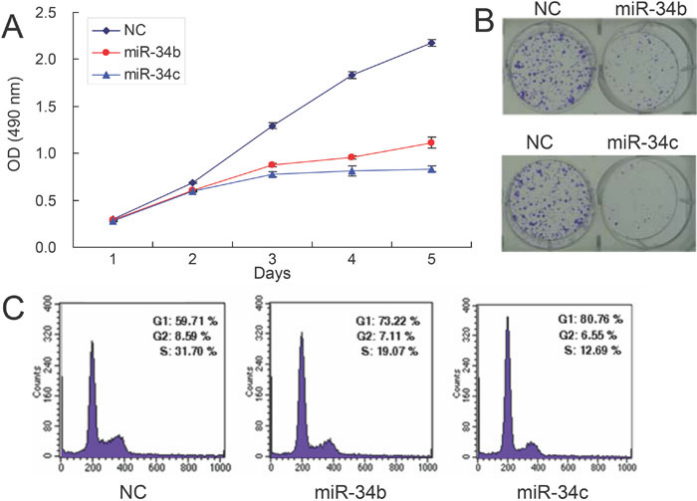
Ectopic microRNA-34 b/c inhibited SP6.5 cell proliferation. **A**: MTS cell proliferation assay was performed on days 1 to 5 as indicated after transfection into SP6.5 cells with either miR-34 b/c mimic or a negative control (NC). Results represent those obtained in three experiments. **B**: SP6.5 cells transfected with miR-34b/c or NC were seeded at low density. Colony formation was observed by staining with crystal violet after seven days. Typical results from three independent experiments are depicted. **C**: SP6.5 cells were transfected with miR-34b/c or NC. After 48 h, cells were collected, stained with propidium iodide, and analyzed by flow cytometry. The most representative results from three independent experiments are shown.

After determining the miR-34b/c expression pattern, we sought to investigate the effects on uveal melanoma cells by restoration of miR-34b/c. SP6.5 cells were transfected with either the miR-34b/c mimic or a negative control. After transfection, the MTS assay was performed to assess cell numbers at days 1 to 5. MiR-34b/c caused a dramatic inhibition of cell proliferation in SP6.5 uveal melanoma cells over a five-day interval (Figure 3A) as compared with the negative control. We detected a decrease of cell numbers in the SP6.5 cell line by day 2 following transfection, and the growth of cells transfected with the miR-34b/c mimic was statistically significantly retarded, as compared with the negative control. A significant reduction in cell number persisted through day 5 (48.85±5.39% decrease for miR-34b and 61.72±3.6% for miR-34c, p<0.01, Figure 3A). With clone formation assay, we were able to visually depict the growth retardation effect of miR-34b/c on SP6.5 cells by crystal violet staining after 7 days of culture (Figure 3B). The mechanism by which miR-34b/c inhibited cell growth was attributed to induction of G_1_ cell cycle arrest. Forty-eight hours after transfection, cell were stained with propidium iodide and analyzed by flow cytometry. In cells transfected with miR-34b, 73.22% of cells accumulated in G_1_ compared with 59.71% of cells for the negative control. In cells transfected with miR-34c, 80.76% of cells accumulated in G_1_ compared with 59.71% of cells for the negative control (Figure 3C). These results indicate that miR-34b/c inhibits cell proliferation and plays an important role in regulating uveal melanoma cell cycle G_1_ phase.

### MicroRNA-34b/c enhanced uveal melanoma cell sensitivity to DOX

**Figure 4:**
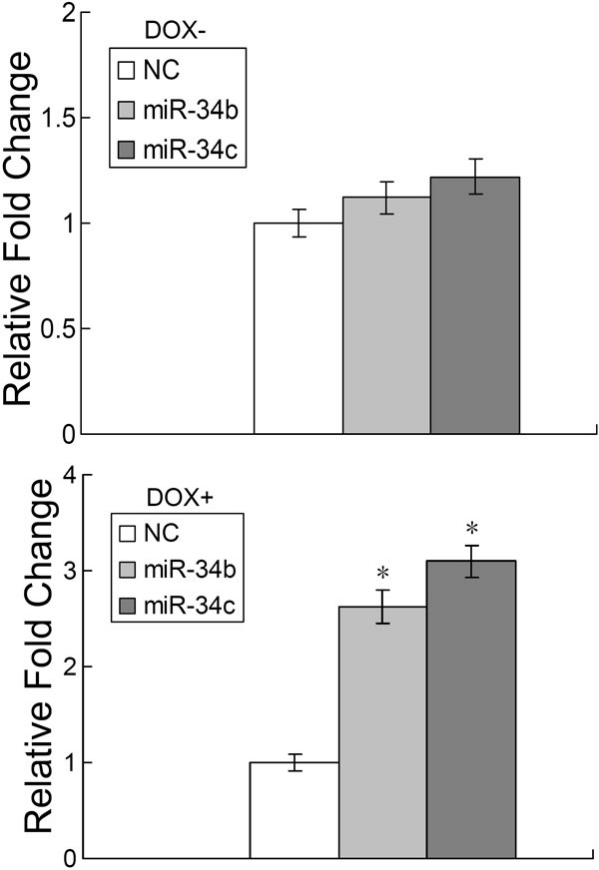
Transfection of microRNA-34 b/c did not induce cell apoptosis but did enhance cell sensitivity to DOX. Caspase 3/7 activity assay was performed on SP6.5 cells transfected with either miR-34b/c or a negative control (NC). Relative caspase 3/7 activity is indicated in comparison to the negative control. Cells were treated without (**A**) or with DOX (**B**). Results represent those obtained in three experiments.

To further characterize the miR-34b/c-mediated inhibition of cell growth, we examined caspase activity to determine if apoptosis was also involved. No significant difference in caspase 3/7 activity was observed between miR-34b/c transfected cells and negative control transfected cells (Figure 4A). Thus, these analyses indicate that miR-34b/c inhibited uveal melanoma cell growth by cell cycle G_1_ arrest rather than by inducing apoptosis. After 48 h, caspase 3/7 activity was significantly increased in miR-34b/c-transfected cells in comparison to negative control after DOX treatment (Figure 4B), which suggested that miR-34b/c enhanced cell sensitivity to DOX.

### MicroRNA-34b/c inhibited uveal melanoma cell migration

**Figure 5:**
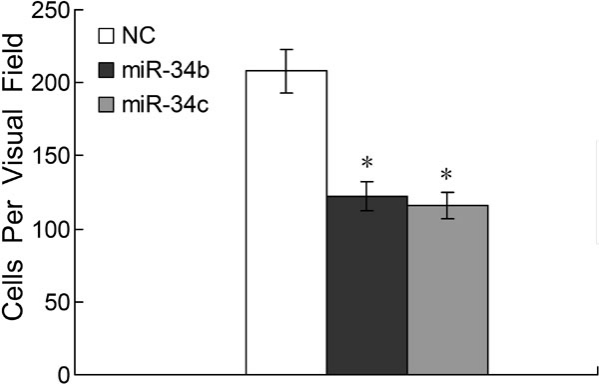
Transfection of microRNA-34 b/c reduced uveal melanoma cell migration. Transwell migration assay of uveal melanoma cell lines was performed. SP6.5 cells were transfected with miR-34b/c or a negative control (NC) for 24 h and plated on cultured inserts in DMEM containing 20 ng/ml of hepatocyte growth factor (HGF) to assess the number of migrating cells. The number of cells that had migrated through the pores was quantified by counting 10 independent visual fields using a 20× microscope objective. *: Differences in cell migration between miR-34b/c and negative control transfected cells were significant, p<0.01.

We next evaluated the effect of miR-34b/c on uveal melanoma cell migration by transwell migration assay. Following transfection with either miR-34b/c mimic or a negative control, SP6.5 cells were seeded on cultured inserts and the ability of cells to migrate to the underside of the inserts was assessed in the presence of HGF. As shown in Figure 5, the migration of cells transfected with miR-34b/c was significantly inhibited, as compared with negative control (208±15 for NC, 122±10 for miR-34b, 116±9 for miR-34c, n=3 each, p<0.01). Therefore, the introduction of miR-34b/c caused reduced cell migration in response to HGF.

### c-Met was a target of microRNA-34b/c

**Figure 6:**
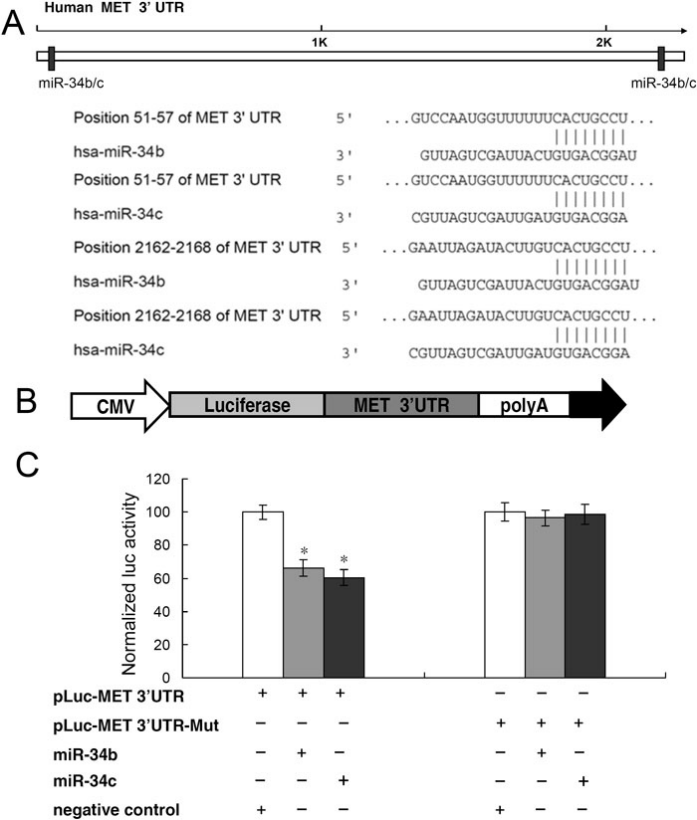
c-Met was a target of microRNA-34 b/c. **A**: Two specific binding sites of miR-34b/c in the *c-Met* 3′ untranslated region (UTR) was marked with black color. Alignment between the predicted miR-34b/c target sites and miR-34b/c, the common 8 bp seed sequence for miR-34b/c:mRNA (mRNA) pairing is shown. **B**: Design of the pMIR luciferase reporter constructs, containing c-Met 3′ UTR, which was used to verify the putative miR-34b/c binding sites. **C**: SP6.5 cells were cotransfected with miR-34b/c, pLuc-MET 3′ UTR, and a pRL-SV40 reporter plasmid. The luciferase activity was measured after 24 h. Values are presented as relative luciferase activity after normalization to *Renilla* luciferase activity. *: Differences in luciferase activity between miR-34b/c and negative control transfected cells were significant, p<0.01.

To explore the molecular mechanisms underlying miR-34b/c mediated cell proliferation and migration, we used TargetScan for miR-34b/c target prediction. As shown in the Figure 6A, two potential binding sites of miR-34b/c, as well as the miR-34b/c: mRNA pairing model, were predicted in the 3′ UTR of the *c-Met* mRNA (Figure 6A). To examine the specific regulation of c-Met through the two predicted binding sites, we cloned the *c-Met* 3′ UTR into the pMIR-REPORT vector (Figure 6B). As expected, the luciferase activity of the wild-type pLuc-MET 3′ UTR construct was significantly suppressed following the transfection of miR-34b/c into SP6.5 cells, in contrast to the negative control (Figure 4C). Mutations of the two 8 bp binding sites in *c-Met* 3′ UTR completely abolished miR-34b/c-mediated inhibition of luciferase activity (Figure 6C). These results demonstrated that c-Met was a direct target of miR-34b/c.

### Introduction of microRNA-34b/c downregulated c-Met, p-Akt, and cell cycle–related proteins

**Figure 7:**
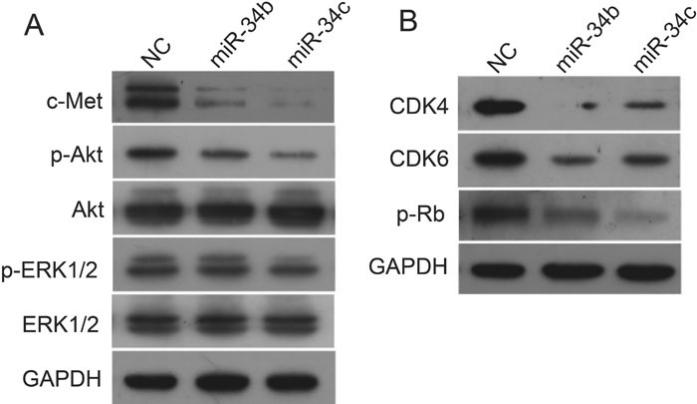
MicroRNA-34 b/c downregulated c-Met, p-Akt and cell cycle proteins in uveal melanoma cells. SP6.5 cells were transfected with miR-34b/c or a negative control. Cell lysates were prepared and used for western blot analysis with c-Met, phosphorylated Akt (p-Akt), total Akt, phosphorylated ERK1/2 (p-ERK1/2), total ERK1/2, CDK4, CDK6, and phosphorylated-Rb (p-Rb) antibodies. Glyceraldehyde-3-phosphate dehydrogenase was used as a loading control. **A**: miR-34b/c downregulated the expression of c-Met and p-Akt. **B**: Cell cycle–related proteins CDK4, CDK6, and p-Rb were downregulated by miR-34b/c.

To confirm that miR-34b/c was indeed responsible for the downregulation of c-Met in uveal melanoma cells, SP6.5 cells were transfected with the miR-34b/c or a negative control. As expected, western blot analysis showed that c-Met expression was dramatically decreased in the cells transfected with miR-34b/c, as compared to negative control (Figure 7). Next, we examined the expression patterns of ERK1/2 and Akt after downregulation of c-Met by miR-34b/c. As shown in Figure 7A, miR-34b/c caused a significant reduction of phosphorylated Akt in SP6.5 cells, but had no obvious effect on ERK1/2 phosphorylation. Neither total Akt nor ERK1/2 expression changed significantly (Figure 7A).

In addition to the effects on c-Met and p-Akt, we also examined the expression of cell cycle–related proteins after transfection with miR-34b/c into SP6.5 cells. As indicated, CDK4, CDK6, and phosphorylated retinoblastoma protein (p-Rb) were dramatically decreased in miR-34b/c-transfected cells (Figure 7B). Thus, miR-34b/c also downregulated cell cycle regulatory proteins in SP6.5 cells (Figure 7B).

## Discussion

Recent evidence has revealed that miRNA plays a central role in tumorigenesis through the regulation of cell proliferation and migration. Until now, however, only several reports about miRNAs in uveal melanoma have been published. miR-34a has been demonstrated to be regulator of *c-Met* oncogene and inhibit cell proliferation and migration [20]. In addition, miR-137 has been found to suppress uveal melanoma cell growth by targeting *MITF* and *CDK6* [21]. Previous studies have identified miR-34b/c as a direct transcriptional target of p53 and an important component of the tumor suppressor network, which operates by modulating cell cycle progression, DNA repair, and apoptosis [10,13]. miR-34b/c is also important in various types of cancer [28-31]. So far, however, little is known about the function of miR-34b/c in uveal melanoma.

To investigate the role of miR-34b/c in uveal melanoma, we first examined miR-34b/c expression in this case. miR-34b/c, expressed in normal uveal melanocytes, is downregulated in both uveal melanoma cells and tumor specimens (Figure 1). Similarly, a previous study has revealed that miR-34a is absent in uveal melanoma cell lines and clinical specimens [20]. The activation of miR-34b/c by 5-aza-dC and TSA indicates that the epigenetic mechanism is responsible for the miR-34b/c downregulation in uveal melanoma cells. In addition, miR-34b/c can be dramatically upregulated by DOX, which provides another way to activate miR-34b/c in uveal melanoma.

Transfection of miR-34b/c into uveal melanoma cells inhibited cell cycle G_1_ arrest and led to significant growth retardation (Figure 3). In contrast to other reports, our results indicated that miR-34b/c did not induce uveal melanoma cell apoptosis directly, but rather enhanced the cell sensitivity to DOX. In addition to cell proliferation and migration, aberrant miRNAs have also been shown to affect tumor cell migration. For example, it has been reported that miR-21 enhanced tumor invasion and metastasis in hepatocellular cancer through regulation of phosphatase and tensin homolog (PTEN) [32], an important tumor suppressor in various types of carcinomas. Here, we demonstrated that miR-34b/c can inhibit uveal melanoma cell migration dramatically in an hepatocyte growth factor (HGF)-dependent fashion.

The tyrosine kinase receptor c-Met is an important proto-oncogene that is upregulated in a variety of cancers [33,34]. c-Met activation, through its ligand HGF, can lead to tumor growth, invasiveness, and metastasis [34-37]. Therefore, we examined the effect of miR-34b/c on c-Met expression and its pathway in uveal melanoma cells. A few miRNAs have been discovered to regulate c-Met expression, such as miR-34a, miR-1/206, and miR-199a* [9,20,38]. In this study, we demonstrated that the other two members of the miR-34 family—miR-34b and miR-34c—play an important role in uveal melanoma cell proliferation and migration with their effects related to the c-Met signaling pathway. In a previous study, it was demonstrated that the PI3K/Akt signaling pathway was involved in HGF-induced migration of uveal melanoma cells through the activation of c-Met [35]. As c-Met expression is directly regulated by miR-34b/c, its downstream effects are similarly altered in uveal melanoma cells.

In addition to regulation of c-Met activity, introduction of miR-34b/c downregulated cell cycle–related proteins, including p-Rb, CDK4, and CDK6. Both CDK4 and CDK6 are important kinases that play an essential role in G_1_ phase progression of the cell cycle [39]. Moreover, CDK4 and CDK6 have been proved to be overexpressed in cutaneous melanoma, which suggests a possible role in melanoma development [40,41]. Rb was first discovered as a tumor suppressor gene, and has a central role in cell cycle regulation and tumorigenesis through control of the G_1_-S transition in proliferating cells [42]. The downregulation of CDK4 and CDK6 (Figure 7B), which downregulates Rb in turn, supports the notion that miR-34b/c can also inhibit cell proliferation through cell cycle protein regulation.

In a previous study of miR-34a in uveal melanoma, it was discovered that miR-34a silence in uveal melanoma, furthermore, miR-34a inhibited cell proliferation and migration by targeting c-Met. However, some details of the function of miR-34a remain unclear. In this study, we also demonstrated that downregulation of miR-34b/c in uveal melanoma, miR-34b/c suppressed cell proliferation and migration by targeting c-Met. Moreover, we have provided strong evidence that miR-34b/c suppressed cell growth by inhibition of cell cycle G_1_ arrest rather than the induction of apoptosis. In addition, we employed multiple approaches to reactivate miR-34b/c, including the use of 5-aza-dC, trichostatin A (TSA), and DOX.

To sum up, our results showed a low level of miR-34b/c expression in uveal melanoma, and provided the approaches to activate miR-34b/c. Restoration of miR-34b/c resulted in the inhibition of cell growth and migration. Specifically, we demonstrated the molecular mechanism of miR-34b/c in the modulation of uveal melanoma cell proliferation and migration. miR-34b/c targeted c-Met, which in turn downregulated the downstream Akt signaling pathway, leading to the inhibition of cell proliferation and migration. In addition, miR-34b/c suppressed cell proliferation via cell cycle proteins CDK4, CDK6, and Rb. Taken together, our findings suggest that miR-34b/c may play an important role in controlling the carcinogenesis of uveal melanoma.
